# Identifying kinesiophobia subtypes and their determinants in patients with hip fractures: a latent profile analysis

**DOI:** 10.3389/fpsyt.2025.1697341

**Published:** 2026-01-26

**Authors:** Yue Cao, Xuefen Lan, Caifu Li, Yingmei Xiang, Qiyao Xu, Ruilin Zhang, Min Li

**Affiliations:** 1 Medicine College, Lishui University, Lishui, Zhejiang, China; 2 Department of Spine Surgery, The First Affiliated Hospital of Lishui University, Lishui People’s Hospital, Lishui, Zhejiang, China

**Keywords:** fear-avoidance, hip fracture, influencing factors, kinesiophobia, latent profile analysis

## Abstract

**Background:**

Limited attention to middle-aged and younger populations has obscured important age-related differences in fear-avoidance mechanisms. Moreover, most existing research on kinesiophobia focuses narrowly on single disease groups and lacks a systematic examination of the multi-dimensional “pain cognition-fear emotion-avoidance behavior” framework. These gaps have led to uniform interventions that overlook diverse fear triggers, hindering precision case and wasting medical resources.

**Objective:**

To identify latent subtypes of kinesiophobia in patients with hip fractures, examine their clinical characteristics, and explore associated factors and intergroup differences, with the aim of informing more targeted intervention strategies.

**Methods:**

This cross-sectional study was conducted between November 2024 and June 2025. Patients with clinically diagnosed hip fractures were recruited through convenience sampling from the orthopedic wards of two tertiary hospitals and two community healthcare centers in Lishui City. Data were collected using the General Information Questionnaire, Tampa Scale for Kinesiophobia (TSK), Self-Rating Scale of Sleep (SRSS), Tilburg Frailty Indicator (TFI), and Visual Analogue Scale (VAS), and subsequently analyzed with SPSS version 27.0. Univariate and multivariate logistic regression analyses were conducted to identify significant predictors of subgroup membership, with the low avoidance type as the reference. Latent profile analysis was conducted using Mplus version 8.3. Among the tested models, the three-class solution (Model 3; AIC = 12738.385, BIC = 13006.411, aBIC = 12784.358, entropy = 0.963) demonstrated the best fit and was selected as the optimal model.

**Results:**

A total of 340 patients with hip fractures were included, with a mean TSK score of 46.68 ± 12.33. Latent profile analysis revealed three distinct kinesiophobia subtypes: low avoidance type, 29.7%, cautious avoidance type, 17.6%, and prominent avoidance type, 52.6%. Logistic regression analysis indicated that academic level, comorbid painful conditions, pain intensity (5.59 ± 3.16), frailty (8.91 ± 4.81), and sleep disturbances (30.99 ± 11.47) were independent predictors of subgroup membership (*P* < 0.05).

**Conclusion:**

This study identified three kinesiophobia subtypes among patients with hip fractures, supporting the relevance of the fear-avoidance model in trauma rehabilitation and providing a basis for tailored intervention strategies (e.g., multimodal therapies for the subtype of significant avoidance kinesiophobia) to optimize rehabilitation outcomes. Future research should investigate the longitudinal trajectory of these subtypes, evaluate the sustained effects of targeted interventions, and examine the generalizability of the model to guide precision rehabilitation care.

## 1 Introduction

Hip fractures are a common type of musculoskeletal trauma and present distinct clinical features across different age groups. Older adults are at the highest risk, largely due to age-related declines in bone quality caused by osteoporosis, which means even minor falls can result in fractures (1). With the global aging population accelerating, the incidence of hip fractures continues to rise. By 2050, the worldwide number of hip fracture cases is projected to reach 6.3 million (2), and in China alone, approximately one million new cases occur each year, with numbers still increasing (3). In contrast, the incidence among middle-aged and younger adults remains relatively low, resulting in limited research focus and challenges in recruiting sufficient sample sizes within practical timeframes (4).

Among patients with hip fractures, older adults represent the vast majority. Numerous domestic studies, such as those by Lu et al. and Lin et al. (5, 6), have focused primarily on hip fracture-related issues in elderly populations. However, research specifically addressing the heterogeneity of kinesiophobia across different age groups remains limited. Notably, Hidaka et al. (7) have highlighted that in elderly patients undergoing hip arthroplasty (7), the associations between kinesiophobia, pain catastrophizing, and quality of life are particularly pronounced. These age-related disparities highlight the need for targeted investigations into kinesiophobia subtypes across diverse populations. Two key limitations are evident. First, as emphasized in a recent systematic review on fear of falling after hip fracture (8), middle-aged and younger patients remain underrepresented, leaving potential age-related differences in fear-avoidance mechanisms largely unexplored. Second, pain not only induces immediate physical discomfort but also progressively undermines psychological resilience, thereby exacerbating patients’ fear of engaging in rehabilitation. Complementing this, a scoping review by Alpalhão et al. (9) on elderly populations suggested that (9), compared with pain itself, kinesiophobia shows a stronger association with frailty and insufficient physical activity. This finding reveals that multiple factors interact in the formation of movement-related fear. This limits the ability to develop interventions that target the specific fear triggers of different subgroups, resulting in generalized approaches that are often ineffective and waste valuable healthcare resources (10, 11).

A review of the literature indicates that factors such as sex and pain intensity are significant predictors of kinesiophobia (12). Pain not only induces immediate physical discomfort but may also progressively compromise psychological well-being, thereby intensifying patients’ fear of engaging in rehabilitation. However, a scoping review focusing on older populations indicates that kinesiophobia is more strongly associated with frailty and physical inactivity than with pain itself, suggesting the multifactorial nature of movement-related fear (9). Additionally, Xie et al. (13) identified marital status as another factor associated with kinesiophobia (13). Based on these findings, this study includes gender, pain intensity, and marital status as independent variables in the multivariate logistic regression analysis. A recent study further extends the fear-avoidance framework by demonstrating that perceived stress mediates the association between pain anxiety and kinesiophobia in surgical patients (14).

The “Fear-Avoidance Model”, first proposed by Lethem et al. (15), provides a core framework for explaining how musculoskeletal pain can progress to chronic pain syndromes. According to this model, fear of pain can lead to two contrasting coping responses: confrontation, where patients face the pain and gradually reduce fear through adaptation, and avoidance, where patients mistakenly associate pain with further harm, reinforcing fear and potentially escalating it into a phobic response. Building on this, Vlaeyen et al. (16) emphasized that individual differences in how pain is perceived and interpreted play a crucial role in triggering pain-related fear and subsequent avoidance behaviors, highlighting the importance of recognizing heterogeneity within the fear-avoidance mechanism (16).

From the perspective of the fear-avoidance model, the pain response and coping behaviors following a hip fracture form a clear psychophysiological chain. Falls commonly cause fractures, and the severe pain that follows activates central sensitization, which can progress to persistent chronic pain (Guidelines for diagnosis and management of geriatric hip fractures, 2022) (17). Chronic pain reinforces the association between movement and pain, triggering a vicious cycle of “pain-related fear” and “movement avoidance”. This cycle often leads to kinesiophobia, a pronounced fear of movement, whose defining features align closely with the “avoidance behavior” described in the fear-avoidance model. Clinically, this manifests as movement avoidance, functional deterioration, and loss of confidence in rehabilitation (18, 19). Evidence shows that fear-driven avoidance behaviors can increase hospital stays by 28% to 40% and raise the risk of readmission by 1.8 times, significantly impeding postoperative functional recovery (20, 21).

Latent profile analysis (LPA) is a person-centered statistical approach that classifies individuals into distinct subgroups based on shared characteristics. For instance, a recent study applied LPA to examine kinesiophobia patterns among patients following cardiac procedures (22), demonstrating the utility of this method in capturing heterogeneous psychological responses to medical interventions, an approach consistent with the analytical framework adopted in the present study. By evaluating multiple model fit indices, such as Akaike information criterion (AIC), Bayesian information criterion (BIC), and entropy, LPA can accurately identify subpopulations with different behavioral and psychological profiles (18). Guided by the fear-avoidance model, this study employs LPA to explore the heterogeneity of kinesiophobia among hip fracture patients across different age groups, from younger to older adults. Specifically, it aims to analyze how subgroups differ in pain cognition, intensity of fear-related emotions, and avoidance behaviors, and to examine how these patterns relate to demographic characteristics, clinical factors, and psychosocial variables. The goal is to clarify the distinct fear-avoidance pathways that contribute to kinesiophobia in different age groups, providing empirical evidence to guide stratified, targeted nursing interventions that can help break the cycle of “pain-related fear and movement avoidance” and ultimately improve patients’ rehabilitation outcomes and quality of life.

## 2 Methods

### 2.1 Design

A cross-sectional study was conducted between October 2024 and June 2025. Postoperative hip fracture patients were recruited via convenience sampling from the orthopedic wards of two tertiary hospitals and two community-based sites in Lishui City. Participants were enrolled within 1–14 days after surgery. This postoperative window was selected because early mobilization is linked to improved functional outcomes. Medically prescribed activity restrictions and postoperative discomfort, however, can limit early activity and may contribute to fear of movement (23).

### 2.2 Setting and participant*s*

Inclusion criteria were: (1) age ≥18 years old; (2) clinically diagnosed hip fracture; (3) clear consciousness and the ability to understand and independently complete the questionnaire and; (4) informed consent and voluntary participation. Exclusion criteria were: (1) severe comorbid heart, brain, kidney, and other major organ diseases; (2) cognitive and behavioral disorders that would interfere with participation; or (3) refusal to participate. The study protocol was approved by the Ethics Committee of Lishui University (No. 2025R003).

The sample size was determined based on the 5th edition of Medical Statistics edited by Yan and Wang (2020) (24). Given that 19 variables were included, the minimum sample size was set at 5–10 times the number of variables (25). Allowing for an expected 20% attrition rate, the minimum required sample size was calculated to be between 119 and 238. Ultimately, 340 participants were recruited for this study.

### 2.3 Instruments

Data were collected using the Tampa Scale for Kinesiophobia (TSK), the Self-Rating Scale of Sleep (SRSS), the Tilburg Frailty Indicator (TFI), the Visual Analog Scale (VAS) for pain, and a researcher-developed socio-demographic questionnaire. The primary outcome variable was the TSK score, while the independent variables included SRSS scores, TFI scores, VAS pain scores and socio-demographic characteristics.

#### 2.3.1 Socio-demographic questionnaire

The socio-demographic questionnaire was developed by the research team based on a review of relevant literature and input from clinical nursing experts (26, 27). It comprised 10 items: gender, age, educational level, marital status, monthly household income, occupation, treatment method, place of residence, living arrangement, and presence of other pain-related conditions.

#### 2.3.2 Tampa Scale for Kinesiophobia

The TSK, originally developed by Kori and Mille (28), was translated into Chinese by Hu (29). It consists of 17 items across two dimensions: activity avoidance and somatic focus (30). Each item is scored on a 4-point Likert scale (1 to 4); items 4, 8, 12, and 16 are reverse-scored. The total score ranges from 17 to 68, with scores above 37 indicating clinically relevant kinesiophobia. The original scale demonstrated good internal consistency (Cronbach’s α = 0.778) and test-retest reliability (0.860). In this study, the Cronbach’s α coefficient was 0.885.

#### 2.3.3 Self-Rating Scale of Sleep

The SRSS was developed by Professor Li Jianming, Executive Director of the Chinese Psychological Health Association and Editor-in-Chief of the *Chinese Journal of Health Psychology* (31). A national standard for the scale was established by a national collaborative research group. The SRSS consists of 10 items, each scored on a 5-point Likert scale (1 to 5), yielding a total score ranging from 10 to 50; higher scores indicate more severe sleep problems. Before establishing the national norm, the scale’s reliability and validity were re-evaluated in a sample of 162 third-year university students, yielding a Cronbach’s α coefficient of 0.6418, r = 0.5625. Given that this study focused on Chinese patients with hip fractures, the SRSS demonstrated greater specificity and suitability. In our sample, the SRSS yielded a Cronbach’s α coefficient of 0.860, indicating good internal consistency and reliable assessment of patients’ sleep status. From the perspective of generalizability, the SRSS provides distinct advantages when applied to specific patient populations in China.

#### 2.3.4 Tilburg Frailty Indicator

The TFI was developed by Gobbens and co-workers at Tilburg University in the Netherlands, based on the integrated frailty model (32), and translated into Chinese by Xi et al. (33). The TFI assesses three dimensions of frailty: physical frailty, psychological frailty, and social frailty. It consists of 15 items scored dichotomously, with a total possible score ranging from 0 to 15; scores of 5 or higher indicate frailty, with higher scores reflecting greater frailty severity. Items 1–8 address the physical dimension, items 9–12 the psychological dimension, and items 13–15 the social dimension. The original scale has a Cronbach’s α coefficient of 0.846; in this study, the Cronbach’s α coefficient was 0.745.

#### 2.3.5 Visual Analogue Scale

The VAS was used to assess pain intensity. In China, the VAS card developed under the supervision of the Pain Society of the Chinese Medical Association is commonly employed (34). The scale uses a 10-cm sliding rulermarked from 0 to 10, where 0 represents “no pain”, and 10 indicates “the worst imaginable pain”. In clinical practice, the marked side is hidden from the patient, who is asked to indicate their pain level along the ruler; medical staff then record the score based on the marked position.

### 2.4 Data collection and quality control

Although there were minor individual differences in the exact timing of the investigations for each research subject, all the surveys were completed within the predefined early recovery window. This approach was adopted to minimize the variability associated with different rehabilitation stages and to control the potential impact of differences in rehabilitation stages on the research results. Prior to questionnaire administration, approval was obtained from the director of the community health service center and the head nurse of the department. The principal investigator (PI) conducted a one-day training session for nurses and nursing postgraduates in the research team, emphasizing adherence to standardized guidelines. After completing the training assessment, a pilot survey was carried out among 80 eligible hip fracture patients recruited from both community and hospital settings. Each questionnaire required approximately 10–15 minutes to complete, and the final version of the instrument was refined based on the pilot survey results. Questionnaires were distributed by trained team members, and participants were asked to complete them independently. For respondents unable to do so, assistance was provided by research staff. Upon on-site collection, questionnaires were checked for missing responses, and any omissions were immediately clarified with participants.

### 2.5 Data analysis

Data analysis was conducted using SPSS version 27.0 (IBM Corp., Armonk, NY, USA). In the logistic regression analyses examining predictors of kinesiophobia subtypes, the dependent variable was the mean score of the two TSK-17 dimensions (treated as a continuous variable). Categorical variables were summarized as frequencies and percentages, while continuous variables following a normal distribution were described by mean ± standard deviation (SD). The chi-square test, independent t-test or Kruskal-Wallis H test was used to compare demographic and clinical characteristics, as well as scale scores, among patients with different kinesiophobia profiles.

LPA, an unsupervised clustering technique designed to explore hidden structures in data and to identify latent subgroups, was conducted using Mplus version 8.3 (Muthén & Muthén, Los Angeles, CA, USA). For model specification, a model with free variances and covariances was selected, which allowed the variances and covariances of TSK items to differ across latent classes, thereby providing greater flexibility in capturing the heterogeneity of kinesiophobia patterns. To determine the optimal number of latent classes, the analysis began with a one-class model, and additional classes were added stepwise (from one to five) until the best-fitting solution was reached (35).

Model fit was evaluated using multiple complementary approaches. First, several information criteria were considered, including the Akaike information criterion (AIC), Bayesian information criterion (BIC), and adjusted BIC (aBIC), where lower values reflect better model fit. Entropy was also examined as an index of classification accuracy, with values closer to 1 indicating more precise assignment of individuals to classes. An entropy value greater than 0.8 was interpreted as good classification accuracy, reflecting a low misclassification rate, high group discrimination, and stable model performance, with most individuals accurately assigned to their respective classes. An entropy value exceeding 0.9 was considered excellent, indicating extremely high classification precision, minimal category overlap, and strong reliability of subgroup identification.

Second, model comparison tests were applied, specifically the Lo-Mendell-Rubin (LMR) likelihood ratio test and the bootstrapped likelihood ratio test (BLRT). Both tests compare the relative fit of the k-class model with the k–1-class model. A statistically significant p-value (<0.05) from either test indicated that the k-class model provided a significantly better fit than the k–1-class alternative.

Following the selection of the optimal model and confirmation of its adequacy, participants were assigned to the latent class for which they demonstrated the highest posterior probability, thereby ensuring that each individual was categorized into the most representative subgroup. After this classification step, multivariate logistic regression analysis was employed to identify factors influencing kinesiophobia in patients with hip fractures, with a p-value of <0.05 considered indicative of statistical significance.

## 3 Results

### 3.1 General characteristics of participants

A total of 376 questionnaires were distributed, of which 340 valid responses were obtained, yielding an effective response rate of 90.43%. Among the 340 respondents, 178 (52.4%) were male and 162 (47.6%) were female. Participants ranged in age from 19 to 79 years, with a mean age of 60.09 ± 5.97 years. The mean TSK score was 46.68 ± 12.33, the average pain score was 5.59 ± 3.16, and the mean frailty score was 8.91 ± 4.81. Additional demographic and clinical characteristics are summarized in **Table 1**.

**Table 1:** Univariate analysis of latent class profiles of kinesiophobia in patients with hip fractures (n = 340).

Item	All (n=340)	Type 1 (n=101)	Type 2 (n=60)	Type 3 (n=179)	Test statistic value	*P*
Gender					1.375^①^	0.503
Male	178(52.4)	48	32	98		
Female	162(47.6)	53	28	81		
Academic level					14.310^①^	<0.001
Junior high school and below	58(17.1)	7	14	37		
Senior high school/secondary vocational school	80(23.5)	24	9	47		
Junior college, undergraduate	115(33.8)	37	12	66		
Graduate student	87(25.6)	33	25	29		
Marital status					5.587^①^	0.067
Single	15(4.4)	1	13	1		
Married	298(87.6)	93	38	16		
Others	16(7.9)	7	9	11		
Monthly family income					5.805^②^	0.055
≤5000	103(30.3)	23	14	51		
5000~7000	131(38.5)	40	15	80		
>7000	106(31.2)	38	31	48		
Occupation					49.089^①^	0.358
Enterprise and public institution	89(23.6)	10	10	5		
Worker	79(21.0)	23	5	51		
Farmer	47(12.5)	42	24	88		
Others	78(20.7)	7	21	36		
Therapy method					6.776^①^	0.034
Expectant treatment	162(47.6)	59	27	76		
Operative treatment	178(52.4)	42	33	103		
Place of abode					0.508^①^	0.776
City	173(50.9)	50	33	90		
rural	167(49.1)	51	27	89		
The resident manner					0.770^①^	0.681
Alone	95(27.9)	29	14	52		
Not alone	245(72.1)	72	46	127		
Whether to combine other diseases that cause pain					21.748^①^	<0.001
Not have	168(49.4)	56	43	69		
Have	172(50.6)	45	17	110		
Age	60.09 ± 5.97	58.57 ± 4.87	63.07 ± 6.31	59.92 ± 6.09	21.498^②^	<0.001
TSK score	46.68 ± 12.33	29.56 ± 4.49	46.12 ± 4.71	56.51 ± 2.69	273.170^①^	<0.001
TIF score	8.91 ± 4.81	4.01 ± 2.35	5.65 ± 3.18	12.76 ± 2.35	230.228^②^	<0.001
SRSS score	30.99 ± 11.47	18.86 ± 3.92	26.05 ± 8.39	39.50 ± 7.35	196.582^①^	<0.001
VAS score	5.59 ± 3.16	2.91 ± 2.51	4.53 ± 2.65	7.46 ± 2.27	139.652^②^	<0.001

Type 1, Low avoidance type; Type 2, Cautious avoidance type; Type 3, Significant avoidance type.

① represents *χ*^2^ test, ② represents *H* test.

### 3.2 Latent profile analysis of kinesiophobia in Patients with hip fractures

A person-centered LPA was conducted on the TSK-17 scores of 340 patients with hip fractures. All 17 items of the TSK-17 were used as manifest indicators to identify latent subgroups of patients based on their kinesiophobia profiles. Latent class models with 1 to 5 categories were constructed. As the number of latent classes increased from 1 to 5, the AIC, BIC, and aBIC values gradually decreased, indicating improved model fit. The model with three latent classes had the highest entropy value (0.963), suggesting excellent classification accuracy. Additionally, both the LMR and BLRT were statistically significant (P < 0.05), indicating that the three-class model provided a significantly better fit than the two-class model.

As shown in **Table 2**, the AIC, BIC, and aBIC values consistently decreased with the addition of more classes; however, the rate of improvement diminished beyond the three-class model. In the four-class model, the entropy value decreased, and the LMR test was not significant (P = 0.075). Similarly, the five-class model showed a further decrease in entropy and a non-significant LMR test (P = 0.162). Based on a comprehensive evaluation of model fit indices, classification accuracy, and interpretability, the three-class model was selected as the optimal solution. In the three-class model, the Akaike Information Criterion (AIC) was 12738.385, the Bayesian Information Criterion (BIC) was 13006.411, the sample-size adjusted Bayesian Information Criterion (aBIC) was 12784.358, and the entropy was 0.963. Information criteria (AIC, BIC, and aBIC) showed substantial improvement from the two- to three-class model, whereas further reductions beyond three classes were marginal. Both the Lo–Mendell–Rubin likelihood ratio test and the bootstrap likelihood ratio test supported the three-class solution over the two-class model (LMR p = 0.028; BLRT p < 0.001), but did not provide additional evidence in favor of models with four or more classes, as the LMR-LRT was no longer statistically significant and the added classes yielded limited incremental improvement. Moreover, the four- and five-class solutions were characterized by the emergence of very small latent classes, which reduced model parsimony and interpretability. In contrast, the three-class model demonstrated high classification accuracy (entropy = 0.963) and well-separated posterior probabilities. Considering statistical fit, classification quality, interpretability, and parsimony, the three-class solution was selected as the optimal model. In this model, the AIC, BIC, aBIC, and entropy values were 12738.385, 13006.411, 12784.358, and 0.963, respectively.

**Table 2:** Fit indices of latent profile analysis for kinesiophobia in hip fracture patients.

Model	L	AIC	BIC	aBIC	Entropy	LMR	BLRT	Class probability
1	-8120.426	16308.852	16439.036	16331.181	–	–	–	
2	-6486.251	13076.503	13275.608	13110.654	0.992	<0.001	<0.001	0.003/0.001
3	-6299.192	12738.385	13006.411	12784.358	0.963	0.028	<0.001	1.000/0.946/0.991
4	-6166.06	12508.121	12845.068	12565.915	0.974	0.0753	<0.001	0.998/0.995/0.996/0.930
5	-6154.523	12521.046	12926.914	12590.662	0.881	0.162	<0.001	0.998/0.996/0.934/0.995/1.000

Model 1 assumed a single latent profile, indicating that all individuals belonged to one homogeneous group with no underlying heterogeneity; Model 2 assumed the presence of two latent profiles in the data.; Model 3 assumed the presence of three latent profiles in the data; Model 4 assumed the presence of four latent profiles in the data; Model 5 assumed the presence of five latent profiles in the data.

### 3.3 Latent profile analysis and classification of kinesiophobia in patients with hip fractures

**Figure 1:**
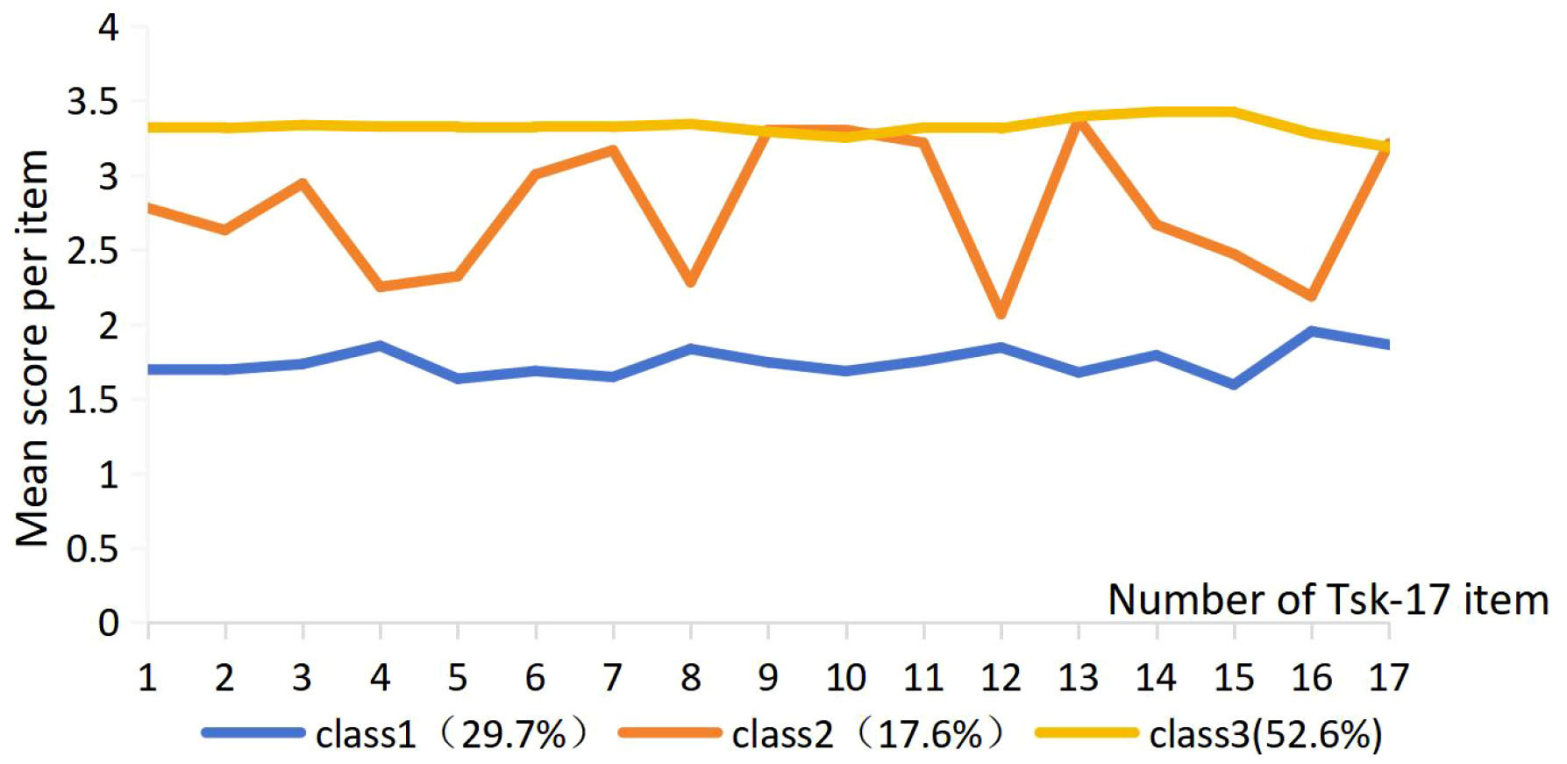
Latent type category of kinesiophobia in hip fracture patients.

**Figure 1** presents the distribution and characteristics of three distinct latent profiles of kinesiophobia identified among patients with hip fractures. These profiles were differentiated based on their kinesiophobia patterns and labeled accordingly to reflect their psychological features. Category 1, labeled the Low avoidance type, included 101 patients (29.7%) with a mean TSK-17 score of 29.56 ± 4.49. This group exhibited consistently low scores across all dimensions of kinesiophobia, indicating minimal fear-related movement avoidance. Profile 2, referred to as the Cautious avoidance type, comprised 60 patients (17.6%) with a mean score of 46.12 ± 4.71, with relatively elevated scores particularly in the motor avoidance dimension, suggesting cautious but controlled behavior. Profile 3, designated the Significant avoidance type, included 179 patients (52.6%) and demonstrated the highest overall kinesiophobia scores, with a mean score of 56.51 ± 2.69. Patients in this group had significantly elevated scores across all dimensions, reflecting pronounced fear and avoidance behaviors. The mean somatic focus scores were 8.71 ± 1.86 in Category 1 (low avoidance subtype), 12.83 ± 2.16 in Category 2 (cautious avoidance type), and 16.68 ± 1.36 in Category 3 (significant avoidance type). The corresponding motor avoidance scores were 20.89 ± 3.38, 34.62 ± 3.44, and 39.89 ± 2.33, respectively (See **Table 3**).

**Table 3:** Comparison of different potential profiles among patients with obsessive-compulsive disorder based on their scores on the TSK-17 questionnaire dimensions.

Latent profile	N	Somatic symptoms, mean ± SD	Activity avoidance, mean ± SD
Low avoidance type	101	8.71 ± 1.86	20.89 ± 3.38
Cautious avoidance type	60	12.83 ± 2.16	34.62 ± 3.44
Significant avoidance type	179	16.68 ± 1.36	39.89 ± 2.33
Total	340	13.64 ± 3.88	33.31 ± 8.80

### 3.4 Univariate analysis of latent kinesiophobia category among patients with hip fractures

Significant differences were observed in the distribution of patients with hip fractures across the three latent kinesiophobia profiles with respect to several demographic and clinical variables, including age, educational attainment, treatment method, and the presence of other pain-related conditions (P < 0.05), as shown in **Table 1**.

### 3.5 Multinomial logistic regression analysis of latent kinesiophobia profiles in patients with hip fractures

Previous studies have shown that higher kinesiophobia scores are associated with poorer compliance with rehabilitation training. To further examine the influence of kinesiophobia on rehabilitation training, multinomial logistic regression analyses were conducted following univariate analysis to identify factors associated with latent kinesiophobia subgroups in patients with hip fractures. The dependent variable was kinesiophobia profile classification, categorized as: 1 = Low avoidance type, 2 = Cautious avoidance type, and 3 = Significant avoidance type. Independent variables included those found to be statistically significant in the univariate analysis. Categorical variables were coded as follows: academic level (Primary school and below = 0, junior high school = 1, High School/Vocational School = 2); treatment method (operative treatment = 0, conservative treatment = 1); presence of comorbid pain-inducing conditions (no = 0, yes = 1). Additionally, continuous scores included pain intensity (VAS score), frailty level (TFI score), and sleep quality (SRSS score). The results of the multivariate logistic regression analysis are summarized in **Table 4**.

**Table 4:** Multivariate logistic regression analysis of latent profiles of kinesiophobia in hip fracture patient.

Variate	Cautious avoidance type and Low avoidance type				Significant avoidance type and Low avoidance type			
	OR	95%CI		P	OR	95%CI		P
		Upper limit	Lower limit			Upper limit	Lower limit	
degree of education								
Primary school and below	6.130	1.194	31.469	0.030	18.432	2.402	141.451	0.005
junior high school	3.683	0.933	14.540	0.063	24.697	3.289	185.432	0.002
High School / Vocational School	0.797	0.239	2.660	0.712	2.142	0.450	10.204	0.339
treatment method								
conservative treatment	0.615	0.247	1.535	0.298	0.609	0.182	2.037	0.421
operative treatment	Ref							
Are there any other diseases that cause pain?								
Not have	3.591	1.216	10.603	<0.001	1.165	1.074	1.264	<0.001
Have	Ref				Ref			
**Age**	1.254	1.135	1.386	<0.001	1.228	1.079	1.398	0.002
**pain score**	1.268	1.031	1.558	<0.001	1.179	0.903	1.538	0.226
**SRSS score**	1.282	1.168	1.406	<0.001	1.400	1.256	1.561	<0.001
**TFI Score**	0.961	0.808	1.144	0.656	1.560	1.266	1.923	<0.001

Multivariate logistic regression analysis (**Table 4**) revealed distinct independent predictors for different types of motion phobia. Compared to the low avoidance group, membership in the cautious avoidance group was independently associated with lower educational attainment (P < 0.05), older age (P < 0.001), higher pain intensity (P < 0.001), higher SRSS scores (P < 0.001), and the absence of other pain-related comorbidities (P < 0.001). Treatment modality was not a significant predictor following adjustment (P > 0.05). Conversely, the significant avoidance group was characterized by lower education level (P < 0.05), older age (P < 0.05), higher SRSS scores (P < 0.001), and higher TFI scores (P < 0.001). Neither pain intensity nor treatment modality showed a significant association for this group (P > 0.05). Although treatment modality was significantly associated with kinesiophobia profiles in univariate analyses (P < 0.05), it did not remain a significant predictor in the multivariate logistic regression model after adjusting for potential confounders (P > 0.05).

Building on Li et al. (36), this study identified three kinesiophobia subtypes in hip fracture patients: Low avoidance type (scores <30), Cautious avoidance type (30-50), and Significant avoidance type (>50). These findings support the applicability of the kinesiophobia avoidance model in trauma rehabilitation and highlight the need for targeted interventions, particularly for significant avoidance type of kinesiophobia patients. Future studies should examine subtype progression, long-term intervention effects, and model generalizability to inform personalized care.

## 4 Discussion

### 4.1 Current status of kinesiophobia in patients with hip fractures

In the present study, 245 out of 340 patients with hip fractures exhibited kinesiophobia, corresponding to a prevalence rate of 72.10%. This rate is considerably higher than the 57.01% reported by Lyu et al. (2024) but lower than the 79.6% reported by Zha et al. (2023) (37, 38). Differences in prevalence may arise from several factors. At the sample level, substantial variation in sample size and selection criteria across studies can introduce bias. For instance, small samples may not adequately represent population characteristics, while restricted selection scopes (e.g., focusing on specific regions, age groups, or disease stages) may yield only partial results. At the level of participant characteristics, heterogeneity in cognitive understanding of kinesiophobia also plays a role: some individuals, limited by insufficient knowledge (38), may lack a systematic awareness of the condition, struggle to recognize their own fear-of-movement symptoms, and thus remain underdiagnosed, contributing to underestimated prevalence in certain studies.

Pain induces physical discomfort and negative experiences, which undermine patients’ confidence in engaging in physical activities. Individuals with low pain acceptance often exaggerate their perception of pain, focus excessively on the possibility of future pain, and display increased sensitivity to anticipated pain, all of which increase the risk of kinesiophobia (39). This hypervigilance can lead patients to subconsciously overestimate the likelihood of re-fracture from environmental factors, with such distress persisting even in safe indoor settings. During rehabilitation, excessive anxiety may evolve into fear of restorative activities such as standing or walking, causing patients to hesitate in their recovery efforts (40). Each attempt at action is suppressed by deep-seated fear of re-fracture, perpetuating a vicious “fear-avoidance” cycle. Within this cycle, fear of movement is continually reinforced, ultimately contributing to a markedly higher incidence of kinesiophobia.

### 4.2 Characteristics of kinesiophobia in patients with hip fractures

LPA identified three distinct subgroups of kinesiophobia: Low avoidance type, Cautious avoidance type, and Significant avoidance type (22). This classification is consistent with previous studies that employed LPA for subgrouping (22). These profiles underscore substantial individual variability in psychological responses to movement-related fear following injury.

#### 4.2.1 Low avoidance type

The Low avoidance type subgroup accounted for 29.7% of the sample, with a score of 29.56 ± 4.49. This subgroup demonstrated a generally low level of movement-related fear, and their score was significantly lower than that of the other two groups (P < 0.001). This quantitative difference supports the relatively weak avoidance tendency of this group toward pain-related activities, aligning with the “confrontation reduces fear” meachanism of the fear-avoidance model. These patients generally adopt rational attitudes toward rehabilitation, actively participate in physical therapy, and maintain positive, cooperative relationships with healthcare providers. Nonetheless, strengthening patient-provider communication and encouraging patients to voice their concerns and emotional needs may further enhance treatment experiences and reinforce their confidence in the rehabilitation process (16).

#### 4.2.2 Cautious avoidance type

This was the largest subgroup, comprising 17.6% of the total study population, with a mean score of 46.12 ± 4.71. Participants in this group demonstrated a tendency toward physical lesion, as indicated by their TSK scores. This behavior appears to be primarily driven by persistent postoperative pain, which significantly impairs daily functioning and reduces overall quality of life. Previous research has identified chronic pain as a major risk factor for delayed or incomplete functional recovery following hip fracture (41). Pain that persists even at rest can exacerbate both physical discomfort and psychological distress (42).

To address these challenges, healthcare providers are advised to conduct regular, dynamic pain assessments and develop individualized treatment plans. Clinical evidence suggests that timely surgical intervention can reduce the likelihood of complications such as avascular necrosis and nonunion (43). Therefore, a combination of appropriate surgical management and comprehensive pain control is essential for improving recovery outcomes in this subgroup.

#### 4.2.3 Significant avoidance type

This subgroup accounted for the largest proportion of participants (52.6%), with a mean score of 56.51 ± 2.69. Their kinesiophobia symptoms were the most severe, as reflected in consistently elevated TSK scores across all items. The mean score of the significant avoidance type group was significantly higher than those of the other two groups (P < 0.001), highlighting their pronounced fear of harm and intense sense of vulnerability when experiencing pain. These fears can lead to a self-reinforcing cycle of anxiety, disrupted sleep, and physical decline (44). A recent study reported that high levels of kinesiophobia are associated with greater pain catastrophizing and poorer quality of life, and may further contribute to a self-reinforcing cycle of anxiety, sleep disturbance, and physical decline (7).

Given the severity of psychological distress in this group, clinical care should prioritize interventions that address both emotional and physical dimensions of recovery. Strategies may include graded exercise therapy to gradually rebuild movement confidence, structured peer support programs to foster social connection and encouragement, and active involvement of family members to provide emotional reassurance. These approaches can collectively enhance patients’ self-efficacy and support a more confident return to rehabilitation.

### 4.3 Factors influencing latent kinesiophobia profiles

Since the introduction of the fear-avoidance model, it has been widely applied in both domestic and international research. Jackowich et al. (45) utilized the fear-avoidance model in a cohort of 263 patients with genito-pelvic sensory disorders (45), demonstrating the model’s effectiveness in identifying cognitive and behavioral factors associated with psychological and functional outcomes. Currently, the fear-avoidance model is widely used in musculoskeletal disorders, supporting its validity in explaining fear-avoidance behaviors.

In the present study, pain severity, sleep disturbances, and frailty were identified as significant factors associated with latent kinesiophobia profile membership. Higher pain scores were positively correlated with elevated levels of kinesiophobia. Pain not only limits physical independence but also negatively impacts psychological well-being, often resulting in emotional distress and diminished social functioning (46, 47). Patients may perceive postoperative pain as a serious threat (48), which promotes movement avoidance and contributes to functional decline.

Among patients in the significant avoidance type, pain, sleep disturbances, and frailty collectively influenced the severity of fear-avoidance behavior. Shou et al. (49) noted that prolonged immobility following hip fracture exacerbates frailty (49), particularly in individuals with pessimistic attitudes or ineffective coping mechanisms (50, 51). Another study by Irwin and Opp (2016) examined the reciprocal regulation between sleep and innate immunity (52). Sleep disturbances are associated with increased production of pro-inflammatory cytokines, such as interleukin-6 (IL-6) and tumor necrosis factor-α (TNF-α). These cytokines play a key role in the body’s inflammatory response and can directly act on nociceptors (pain-sensing neurons), lowering their activation threshold and ultimately increasing pain sensitivity. In patients with hip fractures, sleep disturbances may further exacerbate the injury-induced inflammatory response, intensifying pain perception and potentially promoting avoidance behaviors.

In the present study, among the latent subtypes of kinesiophobia, the frailty score in the cautious avoidance type was 5.65 ± 3.18, which was significantly lower than that in the significant avoidance type (12.76 ± 2.35). In addition, multivariate logistic regression analysis revealed that frailty was not a significant predictor of membership in the cautious avoidance type. Several factors may explain this result. First, psychological status and coping styles appear to play a role (53, 54). Patients in the cautious avoidance type demonstrate intermediate levels of fear of movement and avoidance behaviors, which are not severe enough to establish a vicious ‘fear-avoidance’ cycle. Their perception of rehabilitation is often more rational and objective, which may buffer the negative effects of long-term psychological stress on physiological function, thereby reducing the risk of frailty. Second, these patients tend to maintain relatively intact physiological functions. Compared with the significant avoidance type, they reported significantly lower pain intensity (VAS: 4.53 ± 2.65 versus 7.46 ± 2.27) and fewer sleep disturbances (SRSS: 26.05 ± 8.39 versus 39.50 ± 7.35). As suggested by Cai et al. (55), mild pain and better sleep quality may facilitate greater engagement in rehabilitation training and physical activity, reducing movement resistance and activity avoidance, psychological patterns that could otherwise contribute to physical deconditioning and increased frailty.

In stratified multivariable logistic regression analyses by psychological subgroups, treatment modality (conservative vs. operative) was not an independent predictor of kinesiophobia. In the cautious avoidance group, conservative treatment yielded an OR of 0.615 (P = 0.298), while in the prominent avoidance group, the OR was 0.609 (P = 0.421), with confidence intervals encompassing the null value. These findings indicate no differential association between treatment modality and kinesiophobia risk across subgroups. In contrast, educational attainment consistently emerged as a significant predictor, suggesting that kinesiophobia may be more strongly influenced by psychosocial and cognitive factors than by treatment strategy (56). The largely comparable effects of conservative and operative management on symptom control and rehabilitation trajectories may further attenuate potential differences in fear-related outcomes. Limited consideration of post-treatment rehabilitation experiences and subgroup sample size may have constrained statistical power, warranting further investigation in future studies.Similar observations have been reported in patients with proximal humeral fractures, where conservative and surgical treatments resulted in comparable levels of kinesiophobia prior to structured physiotherapy. These findings suggest that (57), in the early stage of recovery, patients’ fear of movement may be shaped less by the chosen treatment strategy itself and more by shared experiences such as injury-related uncertainty, pain anticipation, and limited functional confidence (57).

### 4.4 Effects of academic level, and pain comorbidities on kinesiophobia classification

This study found that patients with an education level of primary school or below in the significant avoidance type had an 18.432-fold higher likelihood of experiencing kinesiophobia compared to those in the low avoidance type. This suggests that patients with higher educational attainment are more willing to actively acquire disease-related knowledge, have a more comprehensive understanding of the relationship between pain and movement, and demonstrate stronger symptom management skills, which collectively contribute to a lower risk of developing kinesiophobia.

The relationship between pain severity and kinesiophobia is well established. A systematic review by Martinez-Calderon et al. (58) and findings from Tiaho et al. (59) further corroborate a strong correlation between pain intensity and fear of movement (58, 59). Notably, even in the absence of specific pain-related comorbidities, psychological factors, such as anxiety, depression, and psychosocial stress, can elicit disproportionate fear of physical discomfort or re-injury (60). Additionally, physical factors (e.g., fatigue and poor sleep quality) and social-environmental factors (including marital status, family dynamics, and limited social support) significantly contribute to the development and persistence of kinesiophobia.

### 4.5 Implications for clinical practice

Grounded in the fear-avoidance model, this study identified three distinct kinesiophobia types among patients with hip fractures, thereby reinforcing the model’s applicability in trauma rehabilitation (61). The findings highlight the need for individualized assessment and intervention in clinical practice (62). Healthcare professionals should recognize the substantial variability in kinesiophobia across patients and tailor psychological and rehabilitative strategies accordingly, with particular attention to those experiencing high levels of fear and anxiety.

For patients with low avoidance type of kinesiophobia, maintaining active participation in rehabilitation and providing adequate information and support are essential to sustaining progress. For those with cautious avoidance type of kinesiophobia, pain management should be prioritized, coupled with regular dynamic assessments to adjust treatment plans as needed. Patients in the significant avoidance type of kinesiophobia group require comprehensive interventions that address both physical and psychological domains, including graded exercise therapy, peer support, and family involvement, to disrupt the “fear-avoidance” cycle and optimize rehabilitation outcomes.

Overall, enhancing patient education, alleviating emotional distress, and strengthening coping strategies can improve adherence to rehabilitation protocols and promote better functional recovery. Future research should explore the longitudinal evolution of kinesiophobia subtypes, the long-term effectiveness of targeted interventions, and the generalizability of the model to guide precision rehabilitation and individualized care.

## 5 Limitations and suggestions

The study has several limitations that should be acknowledged. First, participants were recruited through convenience sampling from two tertiary hospitals and two community settings in Lishui City, which may have introduced geographical bias. As a result, the findings may not be fully generalizable to hip fracture patients in other regions. Second, although validated instruments such as the TSK and SRSS were employed, no additional psychometric validation (e.g., cross-cultural validity, applicability to specific populations) was performed in this study population. Third, the three latent kinesiophobia profiles identified through LPA were not externally validated, meaning the stability of the classification has yet to be tested in an independent sample. Fourth, functional outcomes related to the identified profiles (e.g., postoperative joint range of motion, activities of daily living scores) were not collected, making it impossible to determine the relationship between kinesiophobia subtypes and actual functional recovery. Finally, in this analysis, fracture mechanism (spontaneous versus traumatic) and baseline depression and anxiety were not included. These factors may influence the assessment of psychological status and limit the comprehensiveness of the model; future studies should consider incorporating them. As a result, these limitations may restrict the clinical applicability of the study’s findings for rehabilitation practice.

Given that postoperative activity restrictions are commonly recommended after hip fracture surgery, patients may be particularly susceptible to developing fear of movement during recovery. Future studies should adopt longitudinal designs to examine the long-term impact of early postoperative activity guidance on the development and progression of kinesiophobia. Moreover, further research is warranted to clarify the temporal evolution of kinesiophobia profiles and to assess the effectiveness of targeted interventions in improving rehabilitation adherence and functional recovery, thereby supporting personalized rehabilitation strategies.

## 6 Conclusion

Grounded in the fear-avoidance model, this study identified three kinesiophobia subgroups among hip fracture patients (low avoidance type, cautious avoidance type, and significant avoidance type) via LPA. Subgroup classification was influenced by pain intensity, frailty, sleep disturbances, and sociodemographic characteristics. These findings highlight the need for tailored interventions that account for patient heterogeneity. Targeted strategies such as patient education, emotional support, and coping skills training may enhance rehabilitation adherence and promote optimal functional recovery.

## Data Availability

The datasets for this article are not publicly available due to concerns regarding participant anonymity. Requests to access the datasets should be directed to the corresponding author.
